# The burden of injury in Brazil, 2003

**DOI:** 10.1590/S1516-31802006000400007

**Published:** 2006-05-04

**Authors:** Vilma Pinheiro Gawryszewski, Eugênia Maria Silveira Rodrigues

**Keywords:** Mortality rate, Morbidity, Violence, Homicide, Accidents prevention, Traffic accidents, Coeficiente de mortalidade, Morbidade, Violência, Prevenção de acidentes, Homicídio, Acidentes de trânsito

## Abstract

**CONTEXT AND OBJECTIVE:**

Since 1980, injuries have been the second biggest cause of death among the Brazilian population. This study aimed to analyze national data on fatal injuries and nonfatal injury hospitalization in Brazil, for 2003.

**DESIGN AND SETTING:**

This was a population-based descriptive study, Brazil, 2003.

**METHODS:**

Data from 126,520 fatal injuries and 733,712 nonfatal injuries seen at public hospitals were analyzed. The data were stratified by sex, age, intent and injury mechanism. Raw and age- and sex-specific rates were calculated per 100,000 individuals.

**RESULTS:**

The raw injury mortality rate was 71.5/100,000 (122.6/100,000 for male and 22.0/100,000 for female). For fatal injuries, the proportions of unintentional and intentional injuries were equal (44.3% and 46.9%, respectively). Homicides were the leading cause, 40.3% overall (28.8/100,000), followed by transport-related deaths, 26.2% overall (17.0/100,000). For nonfatal injuries, the rate was 414.8/100,000 and unintentional injuries were predominant (88.9%). Overall, the leading cause was unintentional falls, accounting for 42.6% of victims treated in public hospitals (176.8/100,000). Transport-related injuries were second: 15.0% overall; 62.0/100,000. Fractures comprised 46.7% of principal diagnoses at hospitals. The injury types in the fatal and nonfatal datasets varied according to sex and age. The highest rates were found among young males and elderly people.

**CONCLUSIONS:**

Injury prevention activities need to be developed. To prevent deaths, homicide has to be addressed. Among hospitalized cases, falls are the most important problem. Traffic-related injuries play an important role in morbidity and mortality.

## Resumo

**CONTEXTO E OBJETIVO:**

A partir da década de 80, a morbimortalidade por causas externas no Brasil se tornou um desafio para a saúde pública. O objetivo deste estudo é analisar os dados de mortes e internações hospitalares no Sistema Único de Saúde devido às causas externas no ano de 2003, último ano disponível.

**TIPO DE ESTUDO E LOCAL:**

Estudo descritivo com base populacional, realizado no Brasil.

**MÉTODOS:**

O universo das 126.520 mortes e 733.712 internações por causas externas foi analisado. Os bancos de dados utilizados foram o Sistema de Informações de Mortalidade (SIM/DATASUS) e o Sistema de Internações Hospitalares (SIH). Os dados foram estratificados por sexo, idade, tipo de causa externa e natureza da lesão. Taxas calculadas por 100.000 habitantes.

**RESULTADOS:**

A taxa de mortalidade por causas externas foi 71,5/100.000 (122,6/100.000 para os homens e 22,0/100.000 para as mulheres). Na mortalidade observou-se um equilíbrio entre a proporção de lesões intencionais e não-intencionais (44,3% e 46,9%, respectivamente). Os homicídios representaram 40,3% do total de mortes (28,8/100.000), seguido pelos acidentes de transporte, 26,2% do total (17,0/100.000). A taxa de morbidade foi de 414,8/100.000, as lesões não-intencionais representaram 88,9%. As quedas foram a maioria (42,6% do total; 176,8/100.000), seguidas pelos acidentes de transporte, 15,0% do total, 62,0/100.000. As fraturas foram o diagnóstico mais comum nas internações.

**CONCLUSÕES:**

Para prevenir mortes, estratégias para reduzir as agressões devem ser desenvolvidas; para prevenir hospitalizações, medidas para redução das quedas devem ser priorizadas.

## INTRODUCTION

Injuries have been shown to account for a significant health burden on many countries around the world.^1^ Each year, injuries account for nearly 1 in 10 deaths worldwide and the mortality rates vary according to the country. Low and middle-income countries suffer disproportionately from reduced life expectancy and poor quality of life.^2^

Since the 1980s, injuries have become a major public concern in Brazil. Two recent reports from the World Health Organization have shown how important this problem is to Brazil, in comparison with other countries worldwide: in the *World report on violence and health*,^3^ Brazil had the third highest homicide rate and, according to the *World report on road traffic injury prevention*,^4^ Brazil was within the top five in terms of traffic-related mortality rates. In 2003, in Brazil, a total of 126, 520 people died because of injuries, accounting for 12.6% of all deaths, and there was a total of 733,712 discharges from public hospitals relating to injuries (6.3% overall).^5^ For each fatal injury, 5.8 other people were hospitalized.^5^ National datasets from hospital emergency departments are not available in Brazil.

Developing an appropriate approach towards injury prevention depends on detailed knowledge about the epidemiology of injuries. In 2003, external causes were in third position among the causes of death for the whole population. Moreover, injuries were the leading cause for people aged between one and 44 years. According to the Brazilian Institute for Geography and Statistics, although life expectancy is increasing in Brazil (71.3 years in 2003), it may have been affected by two or three years because of premature mortality caused by homicides.^6^

## OBJECTIVE

The objective of this study was to analyze national data on fatal injuries and nonfatal injury hospitalization in Brazil for 2003, thereby providing summary data from Brazil to share with people engaged in injury prevention activities in this country and around the world.

## METHODS

This was a population-based descriptive study that had the goal of providing an overview of the national data on fatal injuries and nonfatal injuries treated in public hospitals in Brazil. For mortality, the data are from the Mortality Information System (SIM). This database includes information from all death certificates filled out in the 26 States and Federal District of Brazil. For hospitalization, the data were obtained from the Hospitalization Information System of the Public Health System (SIH/SUS). Both datasets are maintained by Brazil's Ministry of Health.^5^

The case definition adopted for mortality was that this was related to death certificates on which the underlying cause of death was classified in Chapter XX of the International Classification of Diseases, Tenth Revision (ICD-10). Morbidity was taken to relate to the outcomes coded in Chapters XIX and XX of the ICD-10.^7^ With regard to injury intent, *intentional injuries* were considered to be those coded from X60 to Y09, *undetermined intent* was considered to be injuries coded from Y10 to Y34, and *unintentional injuries* were considered to be those with all other codes. The categories used for this analysis were:

- *Transport-related injuries* (V01-V89), which included: *pedestrians* (V01-V09), *pedal cyclists* (V10-V19), *motorcycle riders* (V20-V29) and *motor vehicle occupants* (V40-V79).
- *Other external causes* (W00-X59), which included: *drowning* (W65-W74), *falls* (W00-19), *fire/burns* (X00-X19), *firearm accidents* (W32-W34) and *accidental poisoning* (X40-X49).
- *Intentional self-harm/suicides* (X60-84), which included: *intentional self-poisoning* (X60-X69), *self-hanging, strangulation and suffocation* (X70), and *self-harm by firearm* (X72-X74).
- *Assaults/homicides* (X85-Y09), which included: *assault by firearms* (X93-X95) and *assault by sharp objects* (X99). Injuries relating to legal intervention were included in *homicides/assaults.*
- All other injuries were classified in the *Other causes* group.

For fatal and nonfatal injuries, the variables analyzed were *sex*, *age* and *injury type*. Raw and age- and sex-specific rates were calculated per 100,000 population. For non-fatal injuries, the categories included were the nature of the injury (principal diagnosis), the body region and the lethality of the injury. These were calculated according to age, sex, injury cause and injury intent.

## RESULTS

### Fatal injuries

In 2003, in Brazil, a total of 126,520 people died from an injury: 106,608 were males (84.3%) and 19,740 were females (15.6%). Adolescents and adults aged 15 to 34 years accounted for 49.8% of the injury victims in Brazil. Table 1 shows the frequency of fatal injuries and rates per 100,000 individuals by age group, distributed according to selected types of injuries. The raw injury rate for the whole population was 71.5/100,000 and the male rate (122.6/100,000) was 5.6 times higher than the female rate (22.0/100,000). The mortality rate for young adults aged to 20-24 years reached 121.2/100,000. Although the proportion of deaths among people aged 75 and more was not high (5.3%), the agespecific rate was 180.7/100,000.

**Table 1.** Fatal injuries according to age (number and rate/100,000), Brazil, 2003

Age group (years)	Transport		Pedestrian		Falls		Suicide		Homicide		Total	
	No.	Rate	No.	Rate	No.	Rate	No.	Rate	No.	Rate	No.	Rate
**< 5**	552	3.2	247	1.4	135	0.8	-	-	184	1.1	3,017	17.7
**5 to 9**	719	4.2	385	2.2	80	0.5	6	0.0	100	0.6	1,846	10.7
**10 to 14**	845	4.7	342	1.9	64	0.4	99	0.5	553	3.1	2,678	14.8
**15 to 19**	2,621	14.0	489	2.6	112	0.6	658	3.5	7,943	42.5	13,955	74.7
**20 to 24**	4,241	25.2	604	3.6	190	1.1	1,009	6.0	11,771	69.9	20,416	121.2
**25 to 34**	7,034	23.5	1,197	4.3	502	1.8	1,710	6.1	14,871	53.0	28,772	102.6
**35 to 44**	5,327	22.4	1,380	5.8	786	3.3	1,629	6.9	7,905	33.3	20,314	85.5
**45 to 54**	3,900	23.8	1,155	7.0	754	4.6	1,217	7.4	3,736	22.8	13,145	80.1
**55 to 64**	2,344	22.5	891	8.6	647	6.2	744	7.1	1,666	16.0	7,790	74.8
**65 to 74**	1,649	25.2	802	12.2	779	11.9	469	7.2	703	10.7	5,549	84.8
**75 and +**	1,112	29.8	608	16.3	1,925	51.6	272	7.3	317	8.5	6,748	180.7
**Total**	**30,149**	**17.0**	**8,274**	**4.7**	**6,010**	**3.4**	**7,839**	**4.4**	**50,980**	**28.8**	**126,520**	**71.5**

Analysis combining age and sex showed that the highest injury rate was 221.5/100,000 among males aged 20-24 years. Analysis according to injury intent showed that the proportions of unintentional and intentional injuries were almost equal: 44.3% and 46.9%, respectively. Undetermined injuries accounted for 8.8% of these deaths. Analysis of injury types showed that assaults accounted for 40.3% of all death victims, transport-related causes 26.2%, falls 4.8%, drowning 4.7% and intentional self-harm 6.2%.

Transport-related injuries accounted for 30,149 of the fatal victims, and the highest proportion of these victims was among people aged 20 to 44 years. The raw death rate for the whole population was 17.0/100,000; 28.0/100,000 for males and 6.4/100,000 for females (Table 1). The male/female ratio was 4.4. The fatality rate was greater for people aged 15 and over. Pedestrians accounted for 27.4% of these victims; the age group that showed the highest pedestrian death rates was people aged 65 and over. Motorcyclists accounted for 13.5%.

Fall injuries did not represent a high proportion of the overall number of fatal injuries (6,010 deaths, 4.8% of total); the fatality rate was 3.4/100,000 overall, 4.9/100,000 among males and 2.0/100,000 among females. But the rate for people aged 75 and over was higher, reaching 51.6/100,000 (i.e. 15.2 times higher than the overall death rate due to falls) and the male/female ratio for this age group was 1.0 (Table 1). Analysis of the mechanism for these injuries showed that 45.8% of these cases were classified as unspecified falls; 9.3% was classified as falls from, out of or through buildings or structures.

Suicide victims comprised 7,839 and the suicide rate was 4.4/100,000 overall: 7.2/100,000 among males and 1.8/100,000 among females. The male/female fatality ratio was 7.0. The highest suicide rates were found among people aged 45 to 54 years and 75 and over (Table 1). Analysis of the suicide mechanisms showed that hanging was the leading cause (53.7%), followed by firearms (13.8%).

In Brazil, in 2003, there were 50,980 homicide victims; most of them were among people aged 15 to 44 years. The homicide rates were 28.8/100,000 for the whole population; 54.0/100,000 for males and 4.4/100,000 for females. The male/female ratio was 12.3. The peak was among people aged 20 to 24 years: the rate reached 69.2/100,000 overall and 132.5/100,000 for males in this group.

Firearm gunshots were the most frequent mechanism for homicides: weapons were used in 70.8% of all homicides. But this proportion varied according to sex and age group: it was lower for females (53.6%) than for males (72.2%); for children aged under five it was 19.0% and for people aged 75 and over it was 33.8%. Sharp objects were used in 13.3% of these deaths.

### Nonfatal injuries Nonfatal injuries

In 2003, 733,712 people were admitted to public hospitals in Brazil because of injuries. Table 2 shows the frequencies of nonfatal injuries and rates per 100,000 individuals by age group, distributed according to selected types of injury. The rate was 414.8/100,000 overall; among males it was 594.2/100,000 and among females it was 240.9/100,000. The male/female ratio was 2.5.

**Table 2.** Nonfatal injuries according to age (number and rate/100,000), Brazil, 2003

Age group (years)	Transport		Pedestrian		Falls		Intentional self-harm		Assault		Total	
	No.	Rate	No.	Rate	No.	Rate	No.	Rate	No.	Rate	No.	Rate
**< 5**	3,902	22.8	1,955	11.4	17,440	102.0	332	1.9	998	5.8	42,376	247.9
**5 to 9**	6,695	38.8	3,176	18.4	30,401	176.3	284	1.6	989	5.7	57,556	333.7
**10 to 14**	6,976	38.6	2,697	14.9	27,495	152.2	502	2.8	1,446	8.0	54,328	300.6
**15 to 19**	12,056	64.5	3,341	17.9	23,069	123.4	1,122	6.0	5,729	30.7	64,520	345.2
**20 to 24**	16,816	99.9	3,996	23.7	26,236	155.8	1,342	8.0	8,025	47.7	80,251	476.5
**25 to 34**	23,708	84.5	6,315	22.5	45,368	161.7	2,258	8.1	11,099	39.6	128,126	456.8
**35 to 44**	16,272	68.5	5,363	22.6	40,705	171.3	2,089	8.8	6,714	28.2	102,987	433.3
**45 to 54**	10,257	62.5	4,004	24.4	32,770	199.6	1,293	7.9	3,755	22.9	74,573	454.3
**55 to 64**	5,836	56.0	2,620	25.1	23,585	226.4	557	5.3	1,849	17.7	49,572	475.8
**65 to 74**	4,057	62.0	2,119	32.4	20,078	306.7	322	4.9	985	15.0	38,289	584.8
**75 and +**	3,121	83.6	1,693	45.3	25,546	684.2	204	5.5	830	22.2	41,134	1,101.6
**Total**	**109,696**	**62.0**	**37,279**	**21.1**	**312,693**	**176.8**	**1 0,305**	**5.8**	**42,419**	**24.0**	**733,712**	**414.8**

The proportions of hospitalized injury cases according to age group did not show any marked variation; most of these cases (49.6%) were 20 to 54 years of age. But the rates did vary; the highest rate was among people aged 65 and over.

Analysis according to injury type showed a marked difference in comparison with fatal injuries: among the discharged injury cases, unintentional injury accounted for 88.9% overall. Falls accounted for 42.6%, transport-related 15.6%, assaults 5.8%, self-harm 1.4% and undetermined cause 3.9%.

Nonfatal transport-related injuries comprised 109,691 of the discharged cases and, as found for fatal injuries, the highest proportion of victims was among people aged 15 to 34 years. The rate was 62.0/100,000 overall; 96.7/100,000 for males and 28.4/100,000 for females; the male/female ratio was 3.4. Analysis according to age group showed that the highest rate was among people aged 20 to 24 years, followed by those aged 75 and over. Pedestrians accounted for 34.0% of these injured victims, and 22.4% were motorcyclists. Among pedestrians, the male/female ratio was 1.1, and the distribution according to age group was similar for all nonfatal transport-related discharged cases.

The results from the analysis for motor-cyclists differed from the overall transport-related outcomes. The male/female ratio was higher (6.2), and the highest rates were found among adults aged 20 to 34 years. The lowest rates were found among children and elderly people.

Unintentional falls were the biggest cause of hospitalization, accounting for 312.693 of the discharged cases, i.e. 42.6% overall (Table 2). The rates were 176.8/100,000 overall; 242.9/100,000 among males and 112.7/100,000 among females; the male/female ratio was 2.1. Analysis according to age group showed that the rates were greater for older adults and people aged 75 and over, reaching a fall rate of 684.2/100,000 for this latter age group, in which the hospitalization rate among females was higher than among males. Regarding the mechanisms for fall injuries, 50.6% were due to unspecified falls and 18.8% were due to falls classified as same-level falls due to slipping, tripping or stumbling. These findings for injury mechanisms were similar for males and females but not for age groups: falls from one level to another were more frequent among adults.

Nonfatal assaults accounted for 42,419 discharge cases (5.8% of the total; 24.0/100,000). This was the only type of injury cause in which the number of fatalities was greater than the number of nonfatal victims. Five times more men were hospitalized because of assault (35,255 victims; 40.5/100,000) than women (7,164; 8.0/100,000). As found for homicides, the highest percentage of nonfatal victims and the highest rates were identified among people aged 15 to 34 years. Among the nonfatal assault victims, the proportion of firearm use (30.2% overall) was lower than for fatal assaults. Sharp objects were used in 25.3% and physical force was used in 14.9% of these cases. The use of firearms was more frequent for assaults among people aged 15 to 34 years, whereas among older adults, other means were more frequently used for committing these assaults.

Intentional nonfatal self-harm injuries were the cause of 10,305 discharged cases; the nonfatal rate was 5.8/100,000 overall (7.3/100,000 among males and 4.4/100,000 among females). Male victims (6,328; 7.4/100,000) were 1.5 times more frequently hospitalized due to intentional self-harm injuries than were female victims (4,109; 4.6/100,000); the male/female ratio was 1.7. The age distribution differed from the fatal cases: the rate increased from the 25-34 age group to the 55-64 age group. Moreover, the mechanism differed from that of the fatalities: intentional self-poisoning accounted for 71.9% of these cases, especially exposure to alcohol (X65), pesticides (X68) and drugs, medications and biological substances (X64).

### Hospital mortality rate and principal diagnosis

The hospital mortality rate was 2.7/100,000 overall; 2.8 among males and 2.2 among females. Analysis by age group showed that the highest rates were found among older people; adults aged ≥80 years had the highest rate (7.3). Analysis combining sex and age group showed that the highest rates were among men, for all age groups. Table 3 shows the frequencies, ratios and hospital mortality rates for nonfatal injury cases, distributed according to selected injury types. The hospital mortality rate was almost twice as high among intentional injury cases (4.9) as among unintentional ones (2.7). Also, the rate varied according to the mechanism of injury, and the highest rates were found for intentional or unintentional injuries caused by firearms and intentional self-harm by hanging. The lowest rates were found among unintentional falls, fire/burns and poisoning. These rates were greater for men than for women, for all categories except for fire/burn injuries.

**Table 3.** Nonfatal injuries according to mechanism of injury (number, % and rate/100,000) and hospital mortality rate. Brazil, 2003

Mechanism of injury	Number	%	Rate/100,000 population	Hospital mortality rate
***All unintentional***	652,135	88.9	368.7	2.7
**Transport-related**	109,696	15.0	62.0	4.5
Pedestrian	37,279	5.1	21.1	5.4
Pedal cyclist	10,646	1.4	6.0	2.5
Motorcycle rider	24,604	3.4	13.4	3.5
Motor-vehicle occupant	14,221	1.9	8.0	6.0
Motor-vehicle unspecified	18,068	2.5	10.2	4.5
**Other external causes**	491,640	67.0	278.0	2.0
Falls	312,693	42.6	176.8	1.7
Drowning	2,111	0.3	1.2	2.1
Fire/burns	21,842	3.0	12.4	4.2
Firearms	6,596	0.9	3.7	7.5
Poisoning	9,315	1.3	5.3	2.1
***All intentional***	52,724	7.1	29.8	4.9
**Self-harm**	10,305	1.4	5.8	3.4
Poisoning	7,391	1.0	4.2	2.7
Firearms	549	0.1	0.3	12.9
**Assault**	42,419	5.8	24.0	5.3
Firearms	12,827	1.7	7.3	10.1
Slashing/stabbing	1,522	0.2	0.9	2.8
**Undetermined**	28,853	4.0	16.3	2.5
**Total**	**733,712**	**100.0**	**414.8**	**2.7**

Table 4 shows frequencies, ratios and hospital mortality rates for nonfatal injury cases, distributed according to the nature of the injury. Fractures were the most common principal diagnosis; there were 342,479 victims, representing 46.7% of all discharged injury cases. Analysis according to sex showed that there were more men than women in almost all diagnoses, except for poisoning (4,746 men and 8,049 women). Analysis according to age group showed differences for the age groups at the extremities, in comparison with the others. Among children aged under five, the number of intracranial injuries was similar to the number of fractures in other bones and the proportion of burns/corrosion was higher than in all other age groups. Among people aged 75 and over, fractures were the most important cause of hospitalization. The three diagnoses that showed the highest hospital mortality rates were intracranial injury (10.2), injury of other internal organs (7.9) and fractures of the neck, thorax and pelvis (3.3).

**Table 4.** Nonfatal injuries according to nature of injury (number and %) and hospital mortality rate, Brazil, 2003

Nature of injury	Number	%	Hospital mortality rate
Fracture of skull and facial bones	28,745	3.9	0.7
Fracture of neck, thorax and pelvis	15,424	2.1	3.3
Fracture of femur	58,834	8.0	2.4
Fractures of other bones	240,603	32.8	0.1
Fractures involving multiple regions	1,873	0.3	1.9
Dislocations, sprains and strains	30,043	4.1	0.4
Intracranial injury	94,549	12.9	10.2
Injury of internal organs	26,490	3.7	7.9
Crushing and traumatic amputations	12,552	1.7	2.3
Burns and corrosion	32,387	4.4	2.5
Poisoning by drugs, medications and biological substance	12,795	1.7	2.0
Toxic effects of non-medicinal substances	41,238	5.6	1.8
Maltreatment syndromes	154	0.0	0.7
Other injuries	136,919	18.7	2.7
Information missing regarding nature of injury	1,106	0.2	1.6
**Total**	**733,712**	100.0	**2.7**

## DISCUSSION

Fatal and nonfatal injuries are a major public health problem for all Brazilian residents; in 2003, approximately 157,000 people died as a result of injuries and, for each death, almost six people were treated for an injury in a Brazilian public hospital. This proportion is lower than in the United States, where for every death, around 10 people were hospitalized and/or transferred to a specialized medical care unit, and 178 people were treated and released from an emergency department.^8^ This comparison has to be carefully considered because these numbers may reflect better access to medical care in a developed country, or different policies regarding admissions of injured cases to hospitals.

Violence, particularly homicides, has become a major public concern for Brazilian society. Between 1980 and 2002, the homicide rate more than doubled in Brazil.^9^ The results from the present study showed that assaults had a greater impact on mortality than on morbidity, which is probably an expression of the high proportion of firearms used in assaults. These weapons are more likely to result in death than any other means.^10^ In big cities in Brazil, this proportion may be higher, because studies based on medical examiners’ records have shown that over 90% of homicides are perpetrated with firearms in urban areas such as São Paulo or Recife.^9,11^ In addition, a trend analysis study on firearm mortality in Brazil during the 1990s showed that the homicide mortality rate increased by 27.5% and the firearm homicide mortality rate increased by 72.5%, thus indicating the large contribution of firearms towards the increasing trend of homicide deaths in Brazil.^12^

Brazil has implemented some significant preventive measures at the national level over the past few years to address this problem. One of these was the Disarmament Statute, approved by the Brazilian Congress in December 2003, which is a very strict law that makes it illegal to carry and own firearms, throughout the country, except for the police and armed forces.^13^ In addition, in 2004, a campaign to motivate the Brazilian public to give up their weapons to the judicial authorities was implemented.

Among the unintentional injuries, transport-related events had a great impact on both fatal and nonfatal injuries. The World Health Organization has reported that 88% of deaths due to transport accidents happen in low and middle- income countries, where the numbers of cars are lower than the numbers in developed countries.^4^ Much greater preventive efforts need to be made, in order to reduce the deaths and disabilities associated with motor vehicle injuries among adolescents, young adults and the elderly. Pedestrians ranked the highest among traffic-related injuries, and therefore specific strategies need to be implemented to deal with this problem.

The finding that half of the cases of injuries admitted to public hospitals were due to falls suggests the need for intervention activities to be planned and implemented, in order to reduce this problem, especially among older people. The demographic structure has been changing over recent decades in Brazil, probably due to declines in infant mortality rate and fertility rate.^14,15^ This change has been having a significant impact on all aspects of health statistics and medical care, and there is a very urgent need to address the question of injuries among this age group. There is extensive literature on successful measures for preventing falls among the elderly.^16,17^ Since most of these events have been classified as unspecified falls, there is a need to improve the information and carry out further research, in order to identify how such injuries are occurring and the risk factors involved in such events.

The suicide rate in Brazil is lower than in developed countries.^3^ The age-adjusted suicide rate in the United States in 2001 was 10.7/100,000 people,^18^ i.e. 2.4 times higher than in Brazil. Studies have suggested that it is important to provide support and treatment for people who try to commit suicide and require hospital admission, in order to reduce the risk of further attempts.^19^

The quality of the Brazilian Mortality Information System (SIM) is considered good. However, the data relating to nonfatal injuries should be interpreted with certain restrictions. First, these data represent outcomes from public hospitals and do not include data relating to injured individuals treated in private hospitals. In Brazil, only 24.6% of the whole population has private health insurance (according to the Supplementary National Health Agency; data from 2003).^20^ Public hospitalization is estimated to represent between 88% (northern region of Brazil) and 66% (southern region) of all hospitalization.^20^ Second, because the dataset is very large, it is difficult to obtain nationwide validation and therefore there are no studies available on the quality of the information system. Finally, another potential limitation to the present study is in relation to the high proportion of fall injuries, because assault-related injuries such as intimate partner violence or child maltreatment may have been included in this category. Medical providers may not have asked about or recorded whether or not violence was involved.

Population-based surveillance for emergency departments is also needed in order to monitor injuries and for use in prevention program planning. In Brazil, most emergency departments and health clinics are part of the public health system, but establishing an injury surveillance system remains a challenge. Even in the United States, where twenty-six states have hospital discharge datasets, only nine of them have emergency department datasets available in hospitals.^8^

These results highlight that injuries are one of the most serious health problems in Brazil. The consequences for Brazilians are many and various. Reducing the burden of injuries is a big challenge for public health in Brazil, and understanding the magnitude and characteristics of the problem is the best way to develop injury prevention programs. Brazil has accurate and timely mortality data. Also, timely data on hospitalization is available. Through this, Brazil has been making efforts to promote data analysis in its different states.

## CONCLUSION

The findings from the present study show that the main causes of fatal injuries differ from those that lead to hospitalization. Therefore, in establishing public policies aimed at preventing and monitoring injuries, not only should the mortality data be considered, as usual, but also the hospitalization data needs to be taken into account.
